# Anticholinesterase and Antioxidant Potential of Hydromethanolic Extract of *Ziziphus mucronata* (Rhamnaceae) Leaves on Scopolamine-Induced Memory and Cognitive Dysfunctions in Mice

**DOI:** 10.1155/2019/4568401

**Published:** 2019-10-24

**Authors:** Harquin Simplice Foyet, Eglantine Keugong Wado, Hervé Hervé Ngatanko Abaissou, Emmanuel Acha Assongalem, Oben Kenneth Eyong

**Affiliations:** ^1^Department of Biological Sciences, Faculty of Science, University of Maroua, P.O. Box: 814, Maroua, Cameroon; ^2^Department of Biomedical Science, Faculty of Health Sciences, University of Buea, P.O. Box: 63, Buea, Cameroon; ^3^Department of Organic Chemistry, University of Yaoundé I, P.O. Box 812, Yaoundé, Cameroon

## Abstract

*Ziziphus mucronata* Willd, also known as “buffalo thorn,” belongs to the family Rhamnaceae. Its bark and leaves are used in folk medicine for the treatment of various deficiencies related to nociception, inflammation, mood, and depression. Still, there is a lack of scientific data regarding its potential effect on learning and memory process. The present study was designed to investigate the neuroprotective potential of *Ziziphus mucronata* (ZM) on learning and memory impairment in a scopolamine-induced model of dementia in mice. The phytochemical analysis revealed five cyclopeptide alkaloids (sanjoinines) in the extract from *Ziziphus Mucronata* leaves using LC-HRMS, and the structural characterization of these compounds was determined via MS/MS. Alzheimer-type amnesia was induced by an intraperitoneal injection of scopolamine (1 mg/kg) to mice for 7 consecutive days. ZM (150 mg/kg, 300 mg/kg, and 600 mg/kg) and piracetam (150 mg/kg) were orally administrated to mice daily for a period of 14 days. Memory-related behavioural parameters were evaluated using the radial arm maze task for 7 days, Y-maze, and novel object recognition task. At the end of protocol schedule, animals were sacrificed, and the levels of acetylcholinesterase, malondialdehyde, catalase, and superoxide dismutase were determined in brain homogenates. Histological studies of the hippocampus were subsequently performed. The long-term scopolamine-injected group decreased the spontaneous alternation (Y-maze), the discrimination index, and the time taken to explore the new object (novel object recognition task). These effects were significantly reversed by ZM at all the doses tested. In the radial arm maze task, ZM (300 and 600 mg/kg) significantly decreased the working and reference memory errors when compared with the demented group. Scopolamine-mediated changes in AChE activity were also attenuated by ZM in mice. In addition, extract-treated groups showed a significant increase in the level of CAT and SOD activity and decreased levels of MDA in the mice brains, as compared with the control group. The present study suggests that ZM could have an important role in neuroprotection on this scopolamine-induced model of Alzheimer-type dementia.

## 1. Introduction

Memory refers to the ability to acquire, store, and retrieve information or memory traces, commonly known as “memories.” These memories allow us to handle the present and plan the future [1]. Memory, thus, plays a crucial role in species survival [2]. The identification of neurobiological substrates for memory processes is a fundamental step in their understanding, its ultimate goal being the treatment of human pathologies in which these processes are altered, Alzheimer's disease (AD) being a very current “spearhead.” In this quest, neurobiologists have developed an ever-increasing interest for the cholinergic central system, mainly because there is a positive correlation between the severity of memory deficits observed in Alzheimer's disease and the magnitude in cholinergic neurons loss in the brain of amnesic patients [3]. Thus, a classical approach for the symptomatic treatment of AD is to restore cholinergic functions by prolonging the availability of acetylcholine in the neuronal synapse [4].

Nature provides a new opportunity to manage some neuropsychiatric-related manifestations. In this perspective, several plants have been traditionally used for the management of neurodegenerative disorders and could represent promising candidates for the development of safe, effective, and multitargeted therapies for the treatment of complex neurological disorders such as AD [5].

However, the increased high failure rate of AD drug candidates during clinical trials highlights the complexity of this disease and therefore proves that the origin of memory dysfunction cannot be attributed to cholinergic damage alone [6]. Many neurotransmission systems, including the serotonergic system [7], have been implicated in the modulation of memory function. Additionally, other mechanistic aspects such as oxidative stress, inflammation, and hyperhomocysteinemia are considered important factors of neuronal damage in AD [5]. Therefore, there is a pressing need to develop a multitargeted approach to effectively treat AD.

Scopolamine, a nonselective muscarinic cholinergic antagonist, induces amnesia by decreasing the acetylcholine level and subsequently increasing acetylcholinesterase activity in the brain [8]. Additionally, scopolamine triggers reactive oxygen species (ROS) production, thereby inducing an oxidative stress materialised by an increased lipid peroxidation and deterioration in antioxidant defence mechanisms [9]. Thus, scopolamine has been used as a model in screening antiamnestic drugs [10]. Since ROS mediates neurotoxicity in AD, development of antioxidants as neuroprotective drugs is a potentially beneficial strategy for clinical therapy [11].

*Ziziphus mucronata* Willd (Rhamnaceae), also known as “buffalo thorn,” is a small- to medium-sized tree, with a spreading canopy [12]. Its bark and roots are used in folk medicine for the treatment of various disturbances related to pain, inflammation, and mood [13]. The decoction of *Z. mucronata* leaves is used to treat depression [14]. Moreover, there are evidences that the leaves of this plant have antioxidant activity [15] and anti-acetylcholinesterase activity [16] properties in vitro. These effects could be indicative of their potential use in the treatment of amnesia, a major symptom neurodegenerative disease. However, all these studies were performed *in vitro*, and their transposition to biological systems needs scientific data. To date, no systematic *in vivo* studies on the neuroprotective properties of this plant have been performed. Therefore, this study was designed to investigate the neuroprotective potential of the hydromethanolic extract of *Ziziphus mucronata* leaves on cognitive impairment in scopolamine-induced dementia mice.

## 2. Materials and Methods

### 2.1. Plant Material and Extraction

The fresh leaves of *Ziziphus mucronata* used for this study were harvested from Zokok-Maroua (Far North Cameroon: longitude E 10°57′389”; latitude N 014°24′172”; altitude 428 m). The plant was identified by Dr. Souaré Konsala, a botanist of the Department of Biological Sciences of the University of Maroua. Voucher specimen under number 8868/SER/CAM was deposited in the national herbarium for authentication. The plant name has been checked on the website http://www.theplantlist.org. The leaves were shade-dried at room temperature and protected, and then powdered with an electrical grinder. 1.5 kg of powder was extracted with a mixture of methanol/ water (70 : 30) using a conventional reflux method for 1 h at 50°C in a bathing apparatus. The process was repeated two times and the filtered hydromethanolic extracts were mixed and evaporated using a rotary evaporator 60°C. The concentrated extract was freeze-dried and yielded 208.7 g of lyophilized powder extract (14. 4%).

### 2.2. HPLC-MS Analysis

High-resolution mass spectra (HRMS) were obtained with an Orbitrap Fusion Mass Spectrometer (Thermo Fisher Scientific, USA) equipped with a HESI-II source. Liquid chromatography (LC) was carried out using an Agilent 1290 HPLC system (Agilent Technologies, USA), equipped with a pump, PDA detector, column oven, and autosampler. The separations were performed using a reversed-phase Nucleodur Gravity C18 column, 150 × 2 mm, 1.8 *μ* particle size (Macherey-Nagel GmbH & Co. KG, Germany) with a H_2_O (+0.1% HCOOH) (A)/acetonitrile (+0.1% HCOOH) (B) gradient (flow rate 300 *μ*L/min). Samples were analysed using a gradient program as follows: 95% A isocratic for 0.5 min; linear gradient to 95% B over 9.5 min; after 95% B isocratic for 3 min, the system returned to its initial condition (95% A) within 1 min, and was equilibrated for 1 min. The spectrometer was operated in positive mode (mass range: 70–700 *m/z*) with a resolution of 500 000 at *m/z* 200 and a mass accuracy of ±2 ppm. Selected high-resolution product ion spectra (LC-HR-MS/MS) at a resolution of 60 000 were performed in HCD mode at a normalized collision energy of 15–30%.

### 2.3. Experimental Animals

Male Swiss mice (weighing 20–30 g) were obtained from the breeding facilities of the Laboratory of Biophysics and Biochemistry of the Department of Food Sciences and Nutrition, University of Ngaoundéré, Cameroon. The animals were housed in polyacryl cages (6 animals/ cage) and maintained in a temperature- and light-controlled room (25 ± 2°C, a 12 h cycle). The animals were acclimatized for 7 days. Prior to and after treatment, the animals were fasted for 12 and 7 h, respectively. However, all animals were allowed to drink water *ad libitum*. Mice were treated by following the guidelines of the Cameroonian Bioethics Committee (reg N° FWA-IRB00001954) and in accordance with *NIH-Care and Use of Laboratory Animals* (8th Edition). The protocol was approved by the Ethic Committee of the Faculty of Sciences of the University of Maroua (ref: N° 14/0261/Uma/D/FS/VD-RC).

#### 2.3.1. Chemicals

Scopolamines, acetylthiocholine iodide, 5, 5-dithiobis (2-nitro-benzoicacid) (DTNB), and thiobabituric acid (TBA) were purchased from Sigma-Aldrich, USA. Piracetam was provided by UBC Pharma SA (Belgium). All drugs and extracts were freshly prepared with saline on the day of the experiments. Scopolamine was administered intraperitoneally (i.p.) to the mice, while the extract and piracetam were administered orally (per os, p.o.) in the dosages described below. Control animals received oral administration of 10 ml/kg body of the vehicle.

#### 2.3.2. Experimental Design

**Figure 1:**
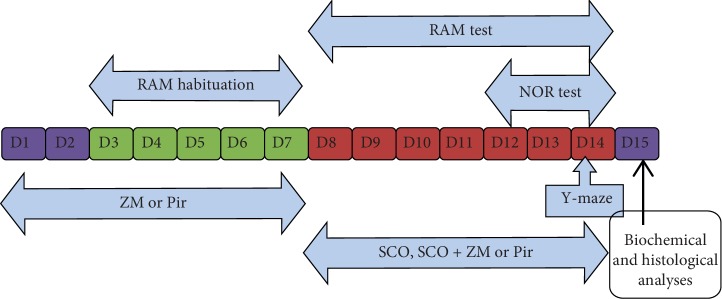
Experimental design procedure for drug administration behavioural study (RAM, NOR, and Y-maze test), biochemical, and histological analysis.

Mice were divided randomly into six groups, with each group comprising 9 mice. Mice in the control group received saline solution (10 ml/kg). Mice in the model group received scopolamine (1 mg/kg, i.p.); mice in the positive control group received scopolamine (1 mg/kg, i.p.) and piracetam (150 mg/kg, *p.o*); mice in the three other groups received scopolamine (1 mg/kg, i.p.) and extract at the doses of 150, 300, and 600 mg/kg. The aforementioned dosage and the duration of treatment were selected using our pilot studies. Scopolamine, piracetam, and extract were all dissolved in saline solution (0.9%). The volume of solution administered to each mouse was 0.1 ml/10 g. The extract or piracetam was administrated once per day throughout the experiment period (14 days). Scopolamine was administered every day from the eighth day to the end of the experiment (Figure 1). Cognitive and memory functions of mice were assessed 30 min after the administration of scopolamine [17].

### 2.4. Behavioural Tests

#### 2.4.1. Radial Arm Maze

Spatial learning and memory were tested using a radial arm maze. Mice were trained and tested in a radial arm task for 7 days as previously described [18]. The radial arm maze used for this study consisted of eight arms, numbered from 1 to 8 (48 cm × 12 cm), extending radially from a central area (32 cm in diameter). The apparatus was placed 50 cm above the floor. Prior to the experiment, the animals were maintained on a restricted feedings schedule designed to keep their body weight at about 85% of the free-feeding level, with water being available *ad libitum*. Before the training began, each mouse was simultaneously subjected to the task and allowed to explore for 5 min and take food freely [19]. The food was initially available throughout the maze, but was gradually restricted to the food cup. The animals were trained for 5 days to run to the end of the arms and consume the bait [20]. The animals were trained for maze task performance by conducting daily training trial for 15 min. Briefly, each animal was placed individually in the centre of the maze and subjected to working and reference memory tasks, in which the same (1, 2, 4, 5, and 7) were baited for each daily training trial. The other three arms (nos. 3, 6, and 8) were never baited throughout the experiment. An arm entry was counted when all four limbs of the rat were within the arm. Measures were made on the number of working memory errors (entering an arm containing food, but previously entered) and reference memory errors (entering an arm that was not baited) [21]. The time taken to consume all five baits was also recorded. Reference memory is regarded as a long-term memory for information that remains constant over repeated trials (memory for the positions of baited arms), whereas working memory is considered as a short-term memory in which the information to be remembered changes in every trial (memory for the positions of arms that had already been visited in each trial) [22]. At the end of each trial, the maze was cleaned with 70% ethanol to eliminate residual odors.

#### 2.4.2. Novel Object Recognition Task

The novel object recognition (NOR) is based on the natural preference for novelty displayed by rodents to assess cognitive alterations in animal models of neurodegenerative disorders [23]. The test was performed using open-field arena in three stages: habituation, familiarization or training, and test phases. Firstly, in order to habituate and reduce the animals' fear of a new environment, mice were exposed to the apparatus with no objects and allowed to freely explore the surroundings for 10 min. Then, the next day, the mice were placed into the open arena with two identical objects (A1 and A2) and were allowed to freely explore the objects for 5 min. Twenty-four hours after the training or the familiarization phase, the mice were returned to the open field in which one of the familiar objects was replaced by the novel object (test phase). Exploratory behaviour was considered only when the mice were sniffing or touching the object with the nose [24]. The time spent exploring the “novel” object (TN) and the time spent exploring the familiar object (TF) were recorded. The recognition memory for each animal was evaluated using the following discrimination index (DI) equation (1):

(1) $$\begin{array}{c} \text{DI}=\frac{\text{TN}-\text{TF}}{\text{TN}+\text{TF}}. \end{array}$$

At the end of each trial, the arena was cleaned with 70% ethanol to eliminate possible scent/trail markers.

#### 2.4.3. Y-Maze Test

The Y-maze task is used to measure spatial working memory through spontaneous alteration in the behaviour in rodents [3]. The Y-maze apparatus consisted of three identical arms (33 cm × 11 cm × 12 cm each) in which the arms are symmetrically separated at 120°. Mice were gently placed at the end of one arm and were allowed to freely explore the Y-maze during 8 min. The sequences of arm entries were recorded during the 5-minute period [25]. Alternation was defined as successive entries into each of the three different arms [2]. The percent alternation is expressed as follows:

(2) $$\begin{array}{c} \%\text{ alternation}=\left(\frac{\text{number of alternations}}{\text{total arm entries}}\right)\times 100. \end{array}$$

The maze was wiped with 70% ethanol between each animal to minimize odor cues. The number of arm entries was used as the indicator of locomotor activity [26].

### 2.5. Brain Sample Preparation

On 15^th^ day of protocol schedule, all mice were anesthetized using chloroform and sacrificed by decapitation. The whole brain was removed for biochemical and histological studies. A subset for biochemical analysis wad weighed and rinsed in the ice-cold isotonic saline solution. Brain tissue samples were then homogenized with 10% (w/v) ice-cold 0.1 M phosphate buffer (pH 7.4). The homogenate was centrifuged at 3000 rpm for 15 minutes at 4°C, and the resultant cloudy supernatant liquid was collected and used for biochemical analysis.

#### 2.5.1. Estimation of Total Proteins

The protein content was measured according to the biuret method [27]. Briefly, 1 mL of Gornall reagent was added to 100 *μ*L of sample. After thirty minutes of incubation at room temperature and in darkness, the absorbance was measured at 540 nm.

#### 2.5.2. Estimation of Brain Acetyl Cholinesterase (AChE) Activity

The AChE activity was assessed using Ellman's method [28]. Briefly, the reaction mixture containing hippocampus homogenate (0.4 ml), 2.6 mL of phosphate buffer (0.1 M, pH 8.0), and 100 *μ*L DTNB was mixed by bubbling air and placing it in a spectrophotometer. Once the reaction milieu was stable, absorbance was read at 412 nm and considered as basal reading. Thereafter, 5.2 *μ*L of ATC was added into the cuvette. The change of absorbance was followed during 2 minutes at 25°C.

#### 2.5.3. Determination of Superoxide Dismutase (SOD)

The SOD activity was measured according to the method described by [29] with a slight modification. Briefly, to 140 *μ*l of homogenate, 1660 *μ*l of carbonate buffer (pH = 10.2) was added, followed by 20 *μ*l of adrenaline (0.3 mM) freshly prepared. The change in optical density/minute was measured at 480 nm against reagent blank. SOD activity was expressed as units/mg of proteins. The blank was made up of 140 *μ*L of distilled water, 1660 *μ*L of carbonate buffer, and 200 *μ*L adrenaline solutions.

#### 2.5.4. Determination of Catalase (CAT)

Catalase activity was measured using the method in [30]. Twenty-five microliters of tissue homogenate was added to 375 *μ*L of phosphate buffer. Then, 100 *μ*L of H_2_O_2_ (50 mM) was introduced into the test tubes. One minute later, 1 ml of potassium dichromate (5%) prepared in 1% glacial acetic acid was introduced into the reaction medium. The tubes were then incubated for 10 minutes in a boiling water bath and then cooled with tap water. Optical densities were recorded at 570 nm [29]. The catalase level in the samples was obtained from a calibration curve previously established. Catalase activity was expressed as *μ* moles of H_2_O_2_ consumed/min/mg of proteins.

#### 2.5.5. Determination of the Level of Malondialdehyde (MDA)

The quantitative measurement of MDA end product of lipid peroxidation in brain homogenate was performed according to the method in [31]. Briefly, 2.0 ml of the tissue homogenate (supernatant) was added to 2.0 ml of freshly prepared 10% w/v trichloroacetic acid (TCA), and the mixture was allowed to stand in an ice bath for 15 min. After 15 min, the precipitate was separated by centrifugation and 2.0 ml of clear supernatant was mixed with 2.0 ml of freshly prepared 0.67% thiobarbituric acid (TBA). The resulting solution was heated in a boiling water bath for 10 min. It was then immediately cooled in an ice bath for 5 min. The absorbance was measured at 532 nm against reagent blank. The concentration of MDA was expressed as nmol/l.

### 2.6. Histopathological Studies

After behavioural and biochemical studies on day 15, a subset of brains from different groups were perfusion-fixed with 4% formaldehyde in 0.1 M phosphate buffer (pH = 7.2). The brains were removed; the hippocampi were carefully isolated according to the recommended technique and postfixed in the same fixative overnight at 48°C. The brains were embedded in paraffin and stained with hematoxylin-eosin. The hippocampus lesions were assessed microscopically at 100X magnification.

### 2.7. Statistical Analysis

All results were expressed as mean ± standard error of the mean (SEM). Data were analysed using one-way analysis of variance (ANOVA). Tukey's posttest was used for multiple comparisons between groups. Data recorded from the acquisition trials of radial arm maze among the groups over a period of seven days were analysed with repeated measures and a multivariate analysis of variance (ANOVA) process of the general linear model. Pearson's correlation coefficient and regression analysis were used to evaluate the connection between behavioural responses and biochemical parameters. Statistical differences were considered significant when the probability *p* was less than 0.5 (*p* < 0.05).

## 3. Results

### 3.1. Phytochemical Screening

**Figure 2:**
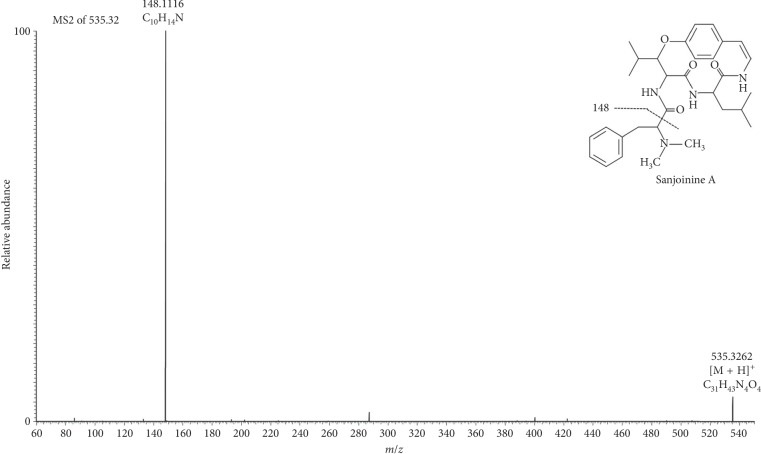
Product ion spectra (MS/MS) of sanjoinine A ([M + H]^+^ 535.3262 *m/z*).

**Figure 3:**
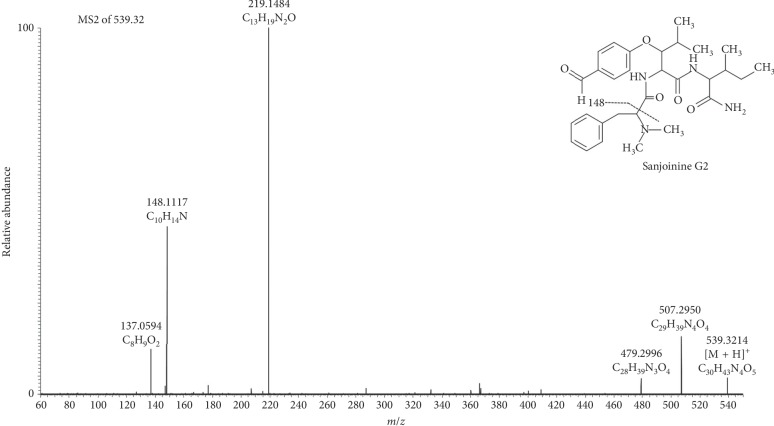
Product ion spectra (MS/MS) of sanjoinine G2 ([M + H]^+^ 539.3214 *m/z*).

Five cyclopeptide alkaloids (sanjoinines) were identified in the extract from *Ziziphus mucronata* leaves using LC-HRMS, and the structural characterization of these compounds was determined via LC-HRMS/MS (Table 1). The presence of these compounds in the family of *Ziziphus* has been previously reported [32]. The product ion spectra of all detected sanjoinine lead to the specific main fragment (*m/z* 148.1116, C_10_H_14_ N]^+^), which was determined as the representative fragmentation of the cyclopeptide side chain (*N*,*N*-demethylphenethylamine) (Figure 2). The MS-MS spectrum of sanjoinine G2 exhibited two further specific fragments, which support the proposed structure (Figure 3).

### 3.2. Effect of Hydromethanolic Extracts of *Z. mucronata* on Spatial Long-Term Memory in the Novel Object Recognition (NOR) Task

**Figure 4:**
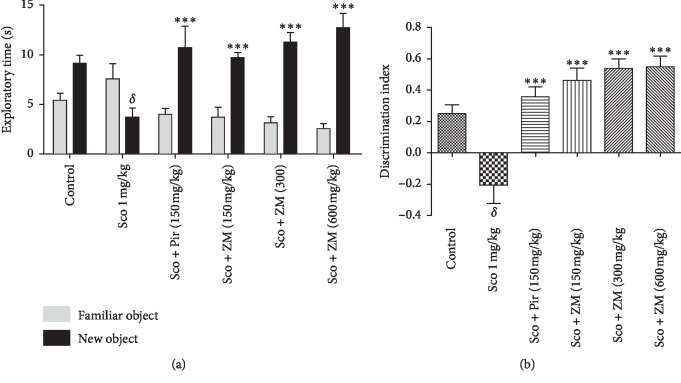
Effect of hydromethanolic extract of *Z. mucronata* leaves and piracetam (Pir) on the exploratory time of the familiar vs. the novel object (a) and the discrimination index (b) after 14 days of treatment on chronically scopolamine (Sco)-treated animal in the object recognition test. Each column represents mean ± S.E.M. of 9 animals. Data analysis was performed using one-way ANOVA followed by Tukey's posttests for discrimination index and two-way ANOVA followed by Bonferroni's posttest for the exploratory time. ^*∗∗∗*^*p* < 0.0001 vs. scopolamine-treated animals. ^*δ*^*p* < 0.001 vs. control group. Scopolamine was administered in the later half for 7 days.

Two-way analysis of variance revealed a significant effect of *Ziziphus mucronata* extract and piracetam (F (5, 72) = 65.84, *p* < 0.0001) on the total amount of time spent exploring the novel object (Figure 4(a)) as compared with the scopolamine-treated group. The discrimination index is commonly used to measure novelty preference in rodents.  Figure 4(b) shows that the scopolamine-treated group displayed poor object recognition when equaled with the normal control group mice as indicated by a lower preference score for the novel object during the test. This observation was significantly reversed (F (5, 53) = 13.46, *p* < 0.0001) by the ZM extract at all the doses tested as well as the piracetam-treated group.

### 3.3. Effect of Hydromethanolic Extracts of *Z. mucronata* on Spatial Working Memory in the Y-Maze Test

**Figure 5:**
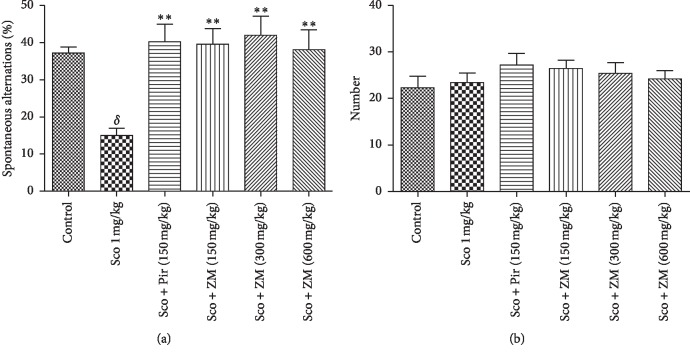
Effect of hydromethanolic extract of *Z. mucronata* leaves and piracetam (Pir) on the locomotor activity (a) and spontaneous alternation percentage (b) after 14 days of treatments on chronically scopolamine (Sco)-treated mice in Y-maze task. Each column represents mean ± S.E.M. of 9 animals. Data analysis was performed using one-way ANOVA followed by Turkey posttest. ^*∗∗*^*p* < 0.001 vs. scopolamine animals, ^*δ*^*p* < 0.001 vs. control group. Scopolamine was administered in the later half for 7 days.

Spontaneous alternation in Y-maze is a specific parameter for the study of spatial working memory [33]. Long-term administration of scopolamine-induced significant reduction in spatial working memory in the Y-maze test (Figure 5(a)) as compared with the normal control group. However, scopolamine-decreased working memory performance was effectively restored by *Ziziphus mucronata* extract at all the doses tested as well as piracetam (Figure 5(b)). The extract-treated groups exhibited significant increase in spontaneous alternation (F (5.30) = 4.999: *p* < 0.001) when compared with the scopolamine-subjected group. Nevertheless, under the same experimental condition, the total number of arm entries was not significantly different among the extract-treated groups and dementia group (Figure 5(b)).

### 3.4. Effect of Hydromethanolic Extracts of *Z. mucronata* on Working and Spatial Reference Memory in the Radial Eight Arms Maze Task

**Figure 6:**
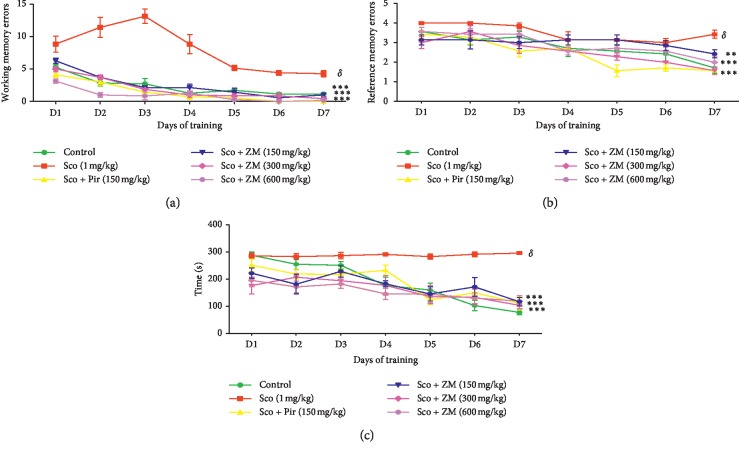
Effects of the hydromethanolic extract of *Z. mucronata* leaves (150, 300, and 600 mg/kg) on the number of working memory errors (a), number of reference memory errors (b), and time taken to consume all five baits (c) in the scopolamine-treated rats during 7 days of training in the radial arm maze test. Values are expressed as mean ± S.E.M. (*n* = 9 animals per group). Data were analysed by using two-way ANOVA followed by Bonferroni post hoc test. ^*∗∗∗*^*p* < 0.0001 vs. scopolamine animals. ^*∗∗*^*p* < 0.001 (Sco vs. Sco + ZM 150 mg/kg). ^*δ*^*p* < 0.001 vs. control group. Scopolamine was administered in the later half for 7 days.

Working memory and reference memory errors are specific parameters for the study of spatial memory in radial maze. Working memory errors were assessed as the number of re-entries into arms already visited during the same trial. The effects of ZM on working memory errors (WMEs) and the reference memory errors (RMEs) are shown in Figures 6(a) and 6(b), respectively. Statistical analyses unveiled a significant decrease ((F5, 252) = 154.50; *p* < 0.0001) in working memory errors in the ZM-treated group as compared with the scopolamine-subjected group. Besides, throughout the test days, the reference memory errors significantly declined ((F5, 216) = 25.1;  *p* < 0.0001) when matched with the amnesic group. Furthermore, two-way ANOVA revealed that the time taken to consume all five baits was significantly decreased in the extract-treated groups ((*F*6, 252) = 35.45: *p* < 0.0001) as compared with the amnesic group (Figure 6(c)).

### 3.5. Effect of Hydromethanolic Extracts of *Z. mucronata* on Brain AChE Activities

**Figure 7:**
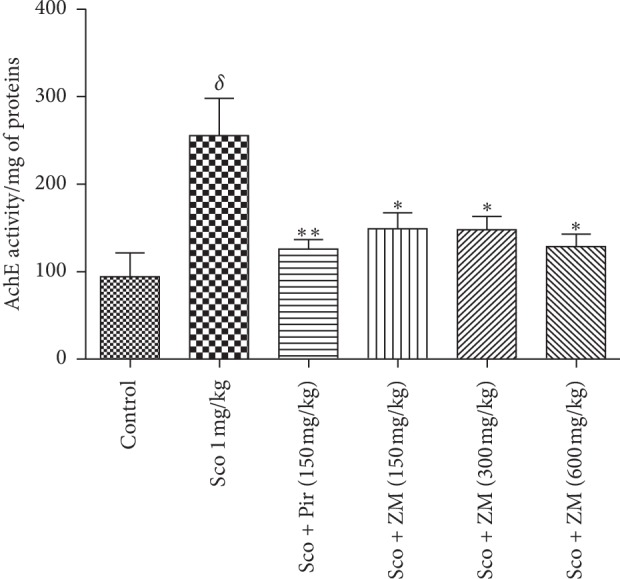
The effect of 14-day oral administration of the hydromethanolic extract of *Z. mucronata* (ZM, 150, 300, and 600 mg/kg) and piracetam (Pir, 150 mg/kg) on AChE activity in mice brain. Values are mean ± S.E.M. (*n* = 6 animals per group). For Turkey's posttest analyses: ^*δ*^*p* < 0.05 vs. Control animals; ^*∗*^*p* < 0.05 Sco vs. ZM-treated group; ^*∗∗*^*p* < 0.001 Sco versus Sco + Pir (150 mg/kg). Scopolamine was administered in the later half for 7days.

As we expected, long-term administration of scopolamine significantly increased the brain AChE activity when compared with the control group. Piracetam significantly lowered (*p* < 0.001) the brain AChE activity (Figure 7) as compared with the scopolamine-induced amnesia group. *Z. mucronata*-treated groups showed also a significant decline (*p* < 0.05) in AChE activity.

### 3.6. Effect of Hydromethanolic Extract of *Z. mucronata* on Brain Lipid Peroxidation and Antioxidant Enzymes

#### 3.6.1. Estimation of Whole Brain Lipid Peroxidation

**Figure 8:**
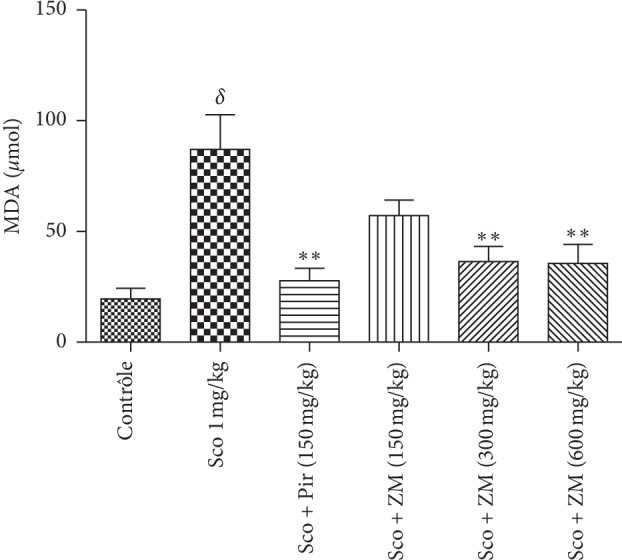
Effect of the hydromethanolic extract of *Z. mucronata* administration on the concentration of MDA. Data were analysed using one-way ANOVA followed by Tukey's posttest. Each column represents mean ± S.E.M. of 6 animals. ^*∗∗*^*p* < 0.01 and when compared with the scopolamine-treated group. ^*δ*^*p* < 0.001 vs. control group. Scopolamine was administered in the later half for 7 days.

Long-term administration of scopolamine significantly elevated the level of lipid peroxidation, as determined through the concentration of MDA, in the brain homogenates when compared with healthy mice. Pretreatment with *Z. mucronata* decreased lipid peroxidation in the whole brain in comparison with the scopolamine group, as indicated by the significant decline in MDA concentration (Figure 8). But this decline in MDA was only significant ((F5, 29) = 7.383 at *p* < 0.0003) in groups that received the extract at dose 300 and 600 mg/kg, as well as the group which received piracetam (150 mg/kg).

#### 3.6.2. Estimation of Catalase Activity

**Figure 9:**
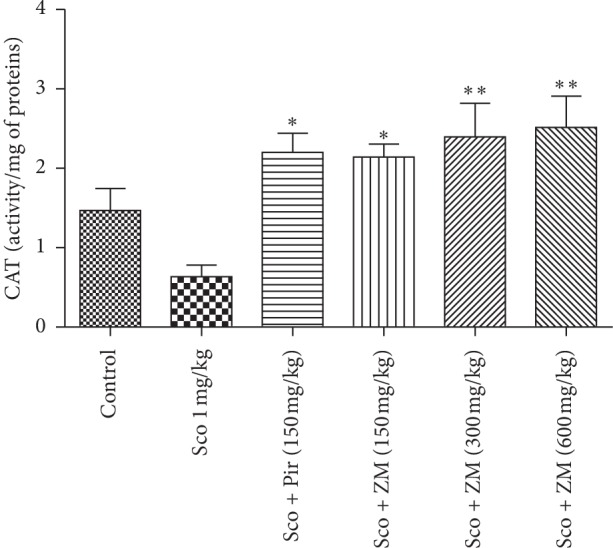
Effect of the hydromethanolic extract of *Z. mucronata* administration on CAT activity. Data were analysed using one-way ANOVA followed by Tukey's post hoc test. Each column represents mean ± S.E.M. of 6 animals. ^*∗*^*p* < 0.05, ^*∗∗*^*p* < 0.01 and when compared with the scopolamine-treated group ^*δ*^*p* < 0.001 vs. control group. Scopolamine was administered in the later half for 7 days.

Results representing the effects of different treatments on catalase activity (Figure 9) show that *Z. mucronata*-treated groups increased the activity of this antioxidant significantly ((F5, 29) = 5.907; *p* < 0.0011) when compared with the dementia group. The piracetam-treated mice also had a significant increase (*p* < 0.05) in catalase activity.

#### 3.6.3. Estimation of SOD Activity

**Figure 10:**
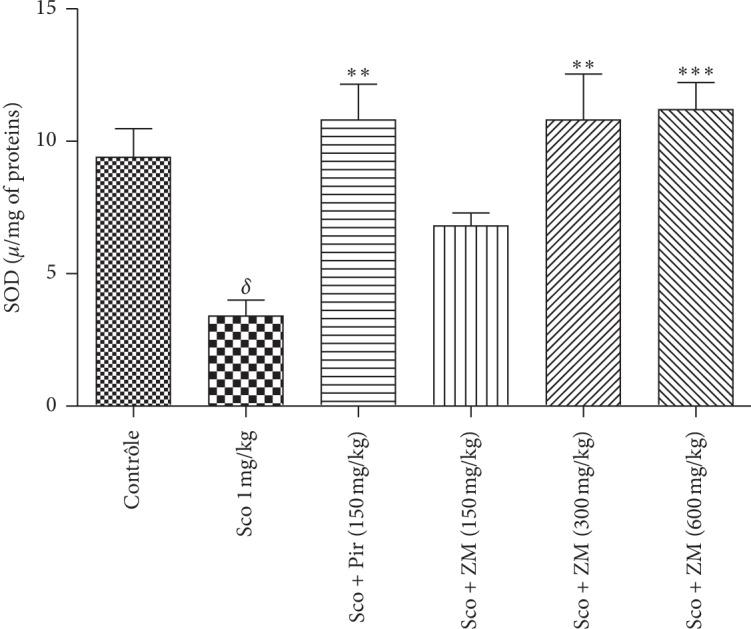
Effect of the hydromethanolic extract of *Z. mucronata* administration on SOD activity. Data were analysed using one-way ANOVA followed by Tukey's post hoc test. Each column represents mean ± S.E.M. of 6 animals. ^*∗∗∗*^*p* < 0.001, ^*∗∗*^*p* < 0.01 when compared with scopolamine-treated group. ^*δ*^*p* < 0.001 vs. control group. Scopolamine was administered in the later half for 7 days.

Regarding SOD activity, the scopolamine-induced dementia group significantly reduced the SOD activity when compared with healthy mice. Pretreatment with *Z. mucronata* at doses 300 and 600 mg/kg significantly increased SOD activity (F (5, 29) = 7.383, *p* < 0.0003) when compared with the scopolamine-induced dementia group. The piracetam-treated group also displayed a significant increase in SOD activity (Figure 10).

**Figure 11:**
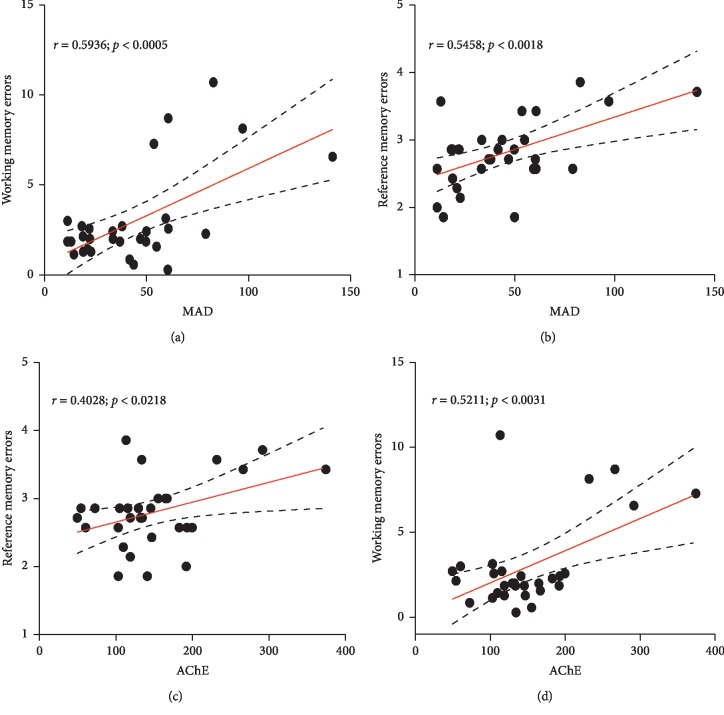
Pearson's correlation between the number of working memory errors versus MAD (a), reference memory errors versus MAD (b), reference memory errors versus AChE (c), and working memory errors versus AChE (d).

More importantly, when linear regression was determined, a signiﬁcant positive correlation between the numbers of working memory errors versus MDA (*r* = 0.5936; *p* < 0.0005) and the number of reference memory errors versus MDA (*r* = 0.5458; *p* < 0.0018) (Figures 11(a) and 11(b) were observed. Furthermore, we found a signiﬁcant positive correlation (*r* = 0.4028; *p* < 0.0218) between acetylcholinesterase activity and reference memory errors on the one hand and on the other hand a strong and significant correlation (*r* = 0.5211; *p* < 0.0031) between acetylcholinesterase activity and working memory errors (Figures 11(c) and 11(d)).

### 3.7. Histopathological Studies

**Figure 12:**
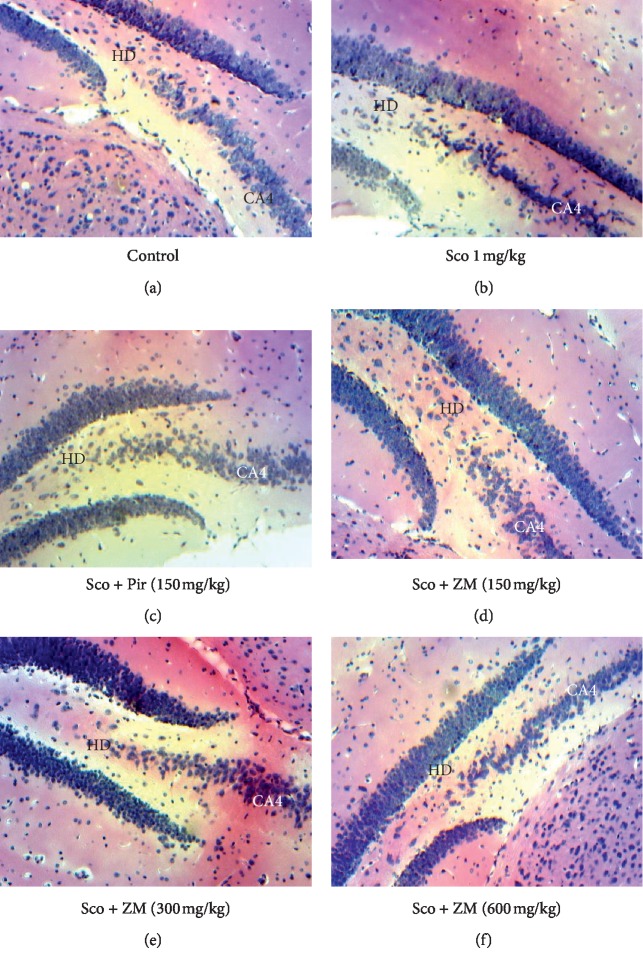
Histopathological studies of hippocampi sections of (a) vehicle-control, (b) scopolamine treated, (c) Sco + piracetam treated, (d) Sco + ZM (150 mg/kg) treated, (e) Sco + ZM (300 mg/kg) treated, and (f) Sco + ZM (600 mg/kg) treated. The hippocampus lesions were assessed microscopically at 100X magnification. CA4: cornu ammonis area 4; HD: hilus of dentate gyrus.

Analysis of histological hippocampi sections at 100X showed that administration of scopolamine decreased cell density in the cornu ammonis (CA4) and the hilus of gyrus dentate, when compared with the normal control group. This observation was reversed by the pretreatment with ZM and piracetam (Figure 12).

## 4. Discussion

The present study showed the nootropic potential of *Ziziphus mucronata* leaves on long-term scopolamine-induced memory and cognitive deficit using Y-maze, radial maze, and novel object recognition in mice.

The Y-maze test is based on the willingness of rodents to explore new environment. Normal animals will prefer to explore a different arm of the maze than the one they visited on their previous entry. This test is mostly used to assess hippocampal-dependent working memory [34]. Therefore, an animal with a memory deficit cannot recall which arm of the maze it has just visited and consequently will exhibit a lower percentage of alternation. The results obtained in this test are clearly showing that seven days' scopolamine-induced dementia significantly decreased the percentage of alternation. This observation was significantly (*p* < 0.001) reversed by the pretreatment with ZM at all the doses, as well as the piracetam. Furthermore, the locomotor activity was not significantly modified by the extract when related with the dementia group. These results suggest that there was not any sedative effect or interference in Y-maze locomotion. Thus, increase in percentage of alternation observed was the result of an improved working memory. Therefore, we hypothesised that ZM extract could ameliorate the working memory. To assess this hypothesis, the working memory was further evaluated in the radial eight arms task. This model is widely used to study spatial working memory and spatial reference in animals. Working memory (WM) is referred to a critical cognitive domain required for the representation of objects or places during goal-directed behaviour [19]. Alternatively, reference memory (RM) is required for temporally stable representations of those objects or places. Chronic administration of scopolamine substantially increased the number of working memory errors, reference memory errors, and the time taken to consume all the five baits. ZM administration improved learning and memory performance as indicated by a significant (*p* < 0.0001) decrease in working memory error and reference memory and an increase in time taken to consume the baits compared with the scopolamine-treated mice. However, the decrease in working memory errors was also observed in the scopolamine-treated group from day 5 to day 7. The decrease in working memory errors is normal as the mice get to learn the position of the baits which is unchanged throughout the experiment. Previous studies revealed that, in animal studies which involve rewarded tasks, working memory errors decreased as the task progressed [35]. Moreover, learning and memory are not only mediated by the cholinergic domain but also by the glutamatergic domain. Besides, although treated with scopolamine, mice still possess the residual memory that can allow them to recall the previously acquired tasks. This may explain why mice though scopolamine-demented could fix the position of the baited arms but to a lower extent as compared with the extract-treated mice.

These results demonstrate the positive effect of ZM on the impaired spatial working and reference memory induced by scopolamine treatment. Moreover, our study reported that, 24 h after the training session, ZM-treated groups significantly increased the exploratory time of the new object instead of the familiar object as compared with the dementia group in the novel object recognition task. Studies with rodents have shown that, for visual object recognition memory, the perirhinal, entorhinal, and inferior temporal cortices, also known as parahippocampal regions of the temporal lobe, are highly important [36]. When some damage exists as it is the case with 7 days of scopolamine injection, the performance in recognition memory tasks will be thus impaired. Most importantly, the discrimination index was significantly increased in the ZM-treated groups when equaled to the scopolamine-treated group in the novel object recognition test. This result corroborates those obtained in the radial arms maze and confirms that ZM could recover long-term memory, as the novel object test is used to assess this type of memory.

In this way, the above exteroceptive behavioural models taken together show that hydromethanolic extract of *Ziziphus mucronata* leaves improves memory and learning in the scopolamine-induced amnesia model in mice. In order to test whether the positive effects of the ZM on memory in scopolamine-demented mice were mediated by the cholinergic system, acetylcholinesterase levels were measured in the mice brain homogenates.

Numerous studies have reported that scopolamine-induced amnesia is mediated by the disruption of cholinergic signalling due to its muscarinic receptor antagonist activity [37]. Scopolamine induces severe loss of cholinergic neurons leading to decrease in levels of acetylcholine in the brain as reported in AD patients [38]. This indicates that the cholinergic system is controlling vital aspect of memory and other cognitive functions. Therefore, acetylcholine esterase inhibitors are the treatments primarily prescribed to patients suffering from AD. A decrease in cognitive function correlates with a decrease in the concentration of acetylcholine at the level of the synaptic cleft, thus implying the primordial role of this neurotransmitter in learning and memory processes [39]. In addition, previous reports showed an increase in AChE activity in rodent brains following scopolamine administration [40]. In the present study, scopolamine significantly elevated AChE activity in the whole brain. This effect was reversed by the pretreatment of ZM. In fact, previous studies have reported that the methanolic extract of ZM leaves possessed an anticholinesterase effect *in vitro* [16]. Thus, the positive effects of this extract on memory can be partially due to the modulation of the cholinergic system. This assertion is verified since the linear regression test showed a significant correlation (*r* = 0.4028; *p* < 0.0218) between acetylcholinesterase activity and working memory errors on the one hand, and on the other hand, a strong and significant correlation (*r* = 0.5211; *p* < 0.0031) between acetylcholinesterase activity and reference memory errors.

The memory impairment in the scopolamine-induced animal model of amnesia is linked in many cases with increased oxidative stress within the brain due to an alteration of brain antioxidant enzymes [41, 42]. The brain is particularly vulnerable to oxidative stress because of its high energy consumption and consequently its high metabolic rate than other cells [43]. Additionally, the brain has high concentration of polyunsaturated fatty acids leading to a self-propagating cascade of lipid peroxidation and molecular destruction [44]. Furthermore, neurons contain relatively low levels of antioxidants for eliminating free radicals [45]. Previous studies have demonstrated that oxidative imbalance and resultant neuronal damage may play a critical role in the initiation and progression of AD [46]. The excessive accumulation of ROS in patients with AD may induce mitochondrial dysfunction. Antioxidant therapy has therefore been suggested for the prevention and treatment of AD [47]. As we expected, the scopolamine dementia group significantly increased lipids peroxidation and decreased catalase activity and SOD activity. ZM-treated groups significantly decreased the MDA concentration and significantly elevated the catalase and SOD activity. In fact, MDA is the by-product of the lipids peroxidation, and its decrease in the ZM-treated groups suggests that ZM could prevent lipids peroxidation. SOD and catalase are both enzymatic antioxidants that protect the cell from reactive oxygen species-related injuries [48]. They play a key role in detoxifying the superoxide anion. The rise in activities of these two enzymes concomitantly with the lowering in MDA concentration suggests that the ZM extract has an antioxidant effect. Moreover, we found a significant positive correlation between the numbers of working memory errors versus MDA and the number of reference memory errors versus MDA. These results could suggest that the increase in behavioural parameters in the radial arm maze tests concomitantly with the increase in MDA could be correlated with the involvement of ZM in neuroprotection against scopolamine-induced oxidative stress generation.

The observed effects could be attributed to the cyclopeptide alkaloids, namely, sanjoinines A, B, F, G1, and G2 found in this plant part. Therefore, the latter could play positive roles in ameliorating cognitive dysfunction observed in this model of scopolamine-induced amnesia by functioning as muscarinic receptor agonists, antioxidants, and acetylcholinesterase inhibitors. Further investigations evaluating the effect of these compounds in scopolamine-induced amnesia are necessary to confirm this finding.

Histological analysis of hippocampi sections at 100X shows that the ZM extract reversed the decrease in cell density (pyramidal cells) in the cornu ammonis and the hilus of gyrus dentate observed in the scopolamine-treated group. CA4 is the continuation of CA3 in the concavity of the dentate gyrus [49]. Hilum of dentate is the complex of pyramidal cell layer and the stratum multiform of the gyrus dentate [50]. The specific function of these structures in memory and cognition still remains to be elucidated. However, it is well documented that the dentate gyrus plays a crucial role in memory formation, storage, and retrieval [51]. Therefore, these results suggest that the ZM extract prevents cell loss and could promote neuronal plasticity. Considering the ethnopharmacological survey and no available data regarding the nootropic effect of ZM *in vivo*, the present study was undertaken to evaluate cognition enhancement, in experimental animal models. The results clearly demonstrate that the ZM extract improves memory and cognitive function by increasing the endogenous acetylcholine level through AChE inhibitors, as well as reducing oxidative stress.

Regarding the limitations of our study, the main aspects we can mention are the absence of an exhaustive database to identify all the family of phytochemical compounds found in this plant and the fact that the bioactive compound was not isolated. So, we intend in our further study to perform a deep insight into the chemistry of this plant part following reference [52].

## 5. Conclusion

The present study shows that ZM treatment ameliorated scopolamine-induced cognitive dysfunction evaluated in the Y-maze, novel object recognition, and radial maze task. Furthermore, the ameliorating effect of ZM was, in part, related to the modulation of cholinergic system function and the reduction of oxidative stress. The observed effects could be attributed to sanjoinines A, B, F, G1, and G2 found in the ZM leaf extract. These findings thus provide further relevance for the potential use of ZM extract as a natural, alternative treatment against psychiatric condition with relevance to Alzheimer disease.

## Figures and Tables

**Table 1:** Cyclopeptide alkaloids identified in leaf extract from *Ziziphus mucronata* plants.

Compound	Chemical formula	tR (min)	[M + H]^+^	[M + H]^+^calcd.	Main product ions created from [M + H]^+^ precursor ion [*m*/*z*] in MS2 spectra
Sanjoinine A	C_31_H_42_N_4_O_4_	5.04; 5.06	535.3262	535.3279	148.1116 [C_10_H_14_ N]^+^
Sanjoinine B	C_30_H_40_N_4_O_4_	4.82	521.3099	521.3122	148.1116 [C_10_H_14_ N]^+^
Sanjoinine F	C_31_H_42_N_4_O_5_	4.64	551.3209	551.3228	148.1116 [C_10_H_14_ N]^+^
Sanjoinine G1	C_31_H_44_N_4_O_5_	4.46	553.3355	553.3384	148.1115 [C_10_H_14_ N]^+^
Sanjoinine G2	C_30_H_42_N_4_O_5_	4.89	539.3207	539.3228	219.1483 [C_13_H_19_N_2_O]^+^, 148.1116 [C_10_H_14_ N]^+^

## Data Availability

No data were used to support this study.
